# Design of Double-Shelled CuS Nanocages to Optimize Electrocatalytic Dynamic for Sensitive Detection of Ascorbic Acid

**DOI:** 10.1186/s11671-020-3278-2

**Published:** 2020-02-18

**Authors:** Tong Yang, Liangliang Tian, Enmin Zhou, Daidong Chen, Yu Lei

**Affiliations:** 1 School of Materials and Energy, Southwest University, Chongqing, People’s Republic of China; 2 Chongqing Key Laboratory of Materials Surface and Interface Science, Chongqing, People’s Republic of China; 3 Chongqing Municipal Key Laboratory of Micro/Nano Materials Engineering and Technology, Chongqing, People’s Republic of China; 4 Research Institute for New Materials Technology, Chongqing University of Arts and Sciences, Chongqing, People’s Republic of China; 5 School of Science, Chongqing University of Posts and Telecommunication, Chongqing, People’s Republic of China

**Keywords:** CuS, Double-shelled nanocages, Cu_2_O template, Electrochemical sensors, Ascorbic acid detection

## Abstract

Although transition metal sulfides have presented prospect in electrochemical sensing, their electrocatalytic performance still cannot meet the demands for practical applications due to the difficulties in mass transport and electron transfer. In this work, double-shelled CuS nanocages (2-CuS NCs) were prepared for enzyme-free ascorbic (AA) sensor through a Cu_2_O- templated method. The unique double-shelled hollow structure displayed large specific surface areas, ordered diffusion channels, increased volume occupying rate, and accelerated electron transfer rate, resulting in enhanced electrochemical dynamic. As a sensing electrode for AA, 2-CuS NCs modified glassy carbon electrode (2-CuS NCs/GCE) exhibited eminent electrocatalytic activity in terms of satisfying sensitivity (523.7 μA mM^−1^ cm^−2^), short response time (0.31 s), and low limit of detection (LOD, 0.15 μM). 2-CuS NCs look promising for analytical sensing of AA in electrochemical sensors thanks to its prominent electrocatalytic kinetics issued from double-shelled hollow porous structure.

## Background

AA plays a key role in biological metabolism for human health. Accurate and fast detection of AA can avoid diseases such as scurvy, diarrhea, and stomach convulsion [1]. A series of methods have already been established to accurately detect AA [2–4]. Thereinto, electrochemical method has attracted lots of attention due to fast response, high sensitivity, simple operation, and low cost. Transition metal materials present great prospect in enzyme-free electrochemical sensors due to their abundant reserves, variable valence states, active redox couples, and accessibility for the detection species [5, 6]. As active materials for electrochemical sensors, transition metal sulfides are a new surge of interest due to their higher electric conductivity compared with transition metal hydroxides or oxides [7].

As we know, the performance of electrochemical sensors closely correlates with the properties of electrocatalysts. Inspired by the structure-activity theory, high-active electrocatalysts can be obtained by controlling their unique morphology and fine structure [8]. Accordingly, researchers have focused on the rational design of electrocatalytic materials with different structures, such as nanosheets, nanorods, nanoplates, nanocubes, and nanospheres. Thereinto, hollow porous structures (HPSs) afford large specific surface areas and sufficient active sites for redox reactions. Moreover, the ultrathin porous shells also shorten distances of ion diffusion or electron transfer [9, 10]. Notably, most of the prepared HPSs are composed of single shells. These single-shelled HPSs commonly suffer from low volume occupying rate (*V*_active materials_/*V*_total_) and limit the further improvement of electrochemical performance [11]. Recently, the attempts to fabricate multi-shelled HPSs have been proposed to circumvent this issue. For example, Shen et al. synthesized NiCo_2_S_4_ ball-in-ball hollow structures with an enhanced specific capacitance of 705 F g^−1^ at 20 A g^−1^ compared with that of single-shelled NiCo_2_S_4_ hollow structures (567 F g^−1^ at 20 A g^−1^) [12]. According to the report of Wang and his coworkers, double-shelled Co_3_O_4_ with higher volume occupying rate exhibited superior specific capacity to the single-shelled one with lower volume occupying rate [11]. Compared with conventional simple single-shelled counterparts, multi-shelled structures with larger surface area and higher volume occupying rate maximize the advantages of HPSs, which means the opportunity to improve physical/chemical properties of active materials and contribute to prominent electrocatalytic performance. Thus, the design of hollow structures with multiple shells is both significant and interesting for electrochemical sensors.

Among transition metal sulfides, CuS is a great candidate for electrochemical sensors, thanks to its effective redox pair of Cu^2+^/Cu^3+^ and metal-like electrical conductivity [13, 14]. In this work, 2-CuS NCs were synthesized through a Cu_2_O-templated method. The prepared 2-CuS NCs have combined advantages of cage-like structure and double-shelled feature, and acquired large specific surface area, desirable porosities and increased volume-occupying rate. As expected, 2-CuS NCs/GCE presented higher electrocatalytic activity in terms of shorter response time (0.31 s), higher sensitivity (523.7 μA mM^−1^ cm^−2^), and lower LOD (0.15 μM) compared with single-shelled CuS nanocages modified GCE (1-CuS NCs/GCE).

## Methods/Experimental

### Reagents

CuCl_2_·2H_2_O, Na_2_S, Na_2_S_2_O_3_·5H_2_O, Na_2_HPO_4_, polyvinylpyrrolidone (PVP, Mw = 40,000), and NaOH were purchased from Chengdu Kelong Chemical Reagent Corporation. Glucose (Glu.), dopamine (DA), lactose (Lac.), fructose (Fruc.), l-ascorbic acid (AA), uric acid (UA), and Nafion solution (5 wt% in mixture of lower aliphatic alcohols and water) were purchased from Sigma-Aldrich without further purification.

### Preparation of Cu_2_O Templates

Cu_2_O templates were obtained according to our previous work [15]. Sixty milliliters of NaOH solution (2 M) was dropped into the stirred CuCl_2_·2H_2_O (600 ml, 0.01 M) at 55 °C. After 30 min of reaction, 60 mL AA (0.6 M) was added into the above solution. The brick-red products were washed and collected by concentration after 3 h, followed by drying in vacuum at 40 °C for 12 h.

### Preparation of 2-CuS NCs

Briefly, 15 mg of Cu_2_O templates were dispersed into a mixed solution of water and alcohol (15 mL, volume ratio 1:1). After fully stirred, 0.45-mL Na_2_S (0.086 M) was added into the solution. The sulfidation lasted for 30 s, and then Cu_2_O@CuS products were collected by centrifugation. Next, Cu_2_O@CuS products were redispersed into 15 mL of mixed solution of water and alcohol (1:1), and 3 mL Na_2_S_2_O_3_ (1 M) was added to etch Cu_2_O for 1 min. After a repeated sulfidation process for 2 min, Cu_2_O templates were completely etched by Na_2_S_2_O_3_ (1 M) for 1 h. The final products were washed and collected by centrifugation, and then dried in vacuum at 60 °C for 12 h. 1-CuS NCs samples were obtained without a repeated sulfidation process (see FESEM and TEM images in Additional file 1: Figure S1).

### Electrochemical Measurements

All electrochemical measurements were carried out in 0.1 M phosphate solution (PBS) on an electrochemical workstation (μIII Autolab). The modified GCEs, Ag/AgCl, and Pt electrodes were considered as working electrodes, reference electrode, and the counter electrode, respectively. GCEs (*Φ* = 3 mm) were firstly polished by 1, 0.5, and 0.05 μm alumina slurry. Then, the polished GCEs were successively cleaned with diluted HNO_3_, water, and ethanol under ultrasonic. Afterward, 5 mg products (2-CuS NCs or 1-CuS NCs) were dispersed into a mixture of 0.9 mL water and 0.1 mL Nafion. Five microliters of suspension was then dropped onto pretreated GCEs and dried at room temperature. The modified GCEs were denoted as 2-CuS NCs/GCE and 1-CuS/GCE, respectively.

### Apparatus and Instruments

Crystal structures of the samples were characterized by X-ray diffraction (XRD, Rigaku D/Max-2400). The compositions were analyzed by X-ray photoelectron spectroscopy (XPS, ESCALAB250Xi) with the C 1s peaks (284.8 eV) as an internal standard. The morphologies were observed via field emission scanning electron microscope (FESEM, SU8020) and high-resolution transmission electron microscope (HRTEM, FEIF20). Brunauer-Emmett-Teller (BET, Belsort-max) was utilized to analyze the specific surface area and pore structure.

## Results and Discussions

### Characterizations of the Products

**Fig. 1:**
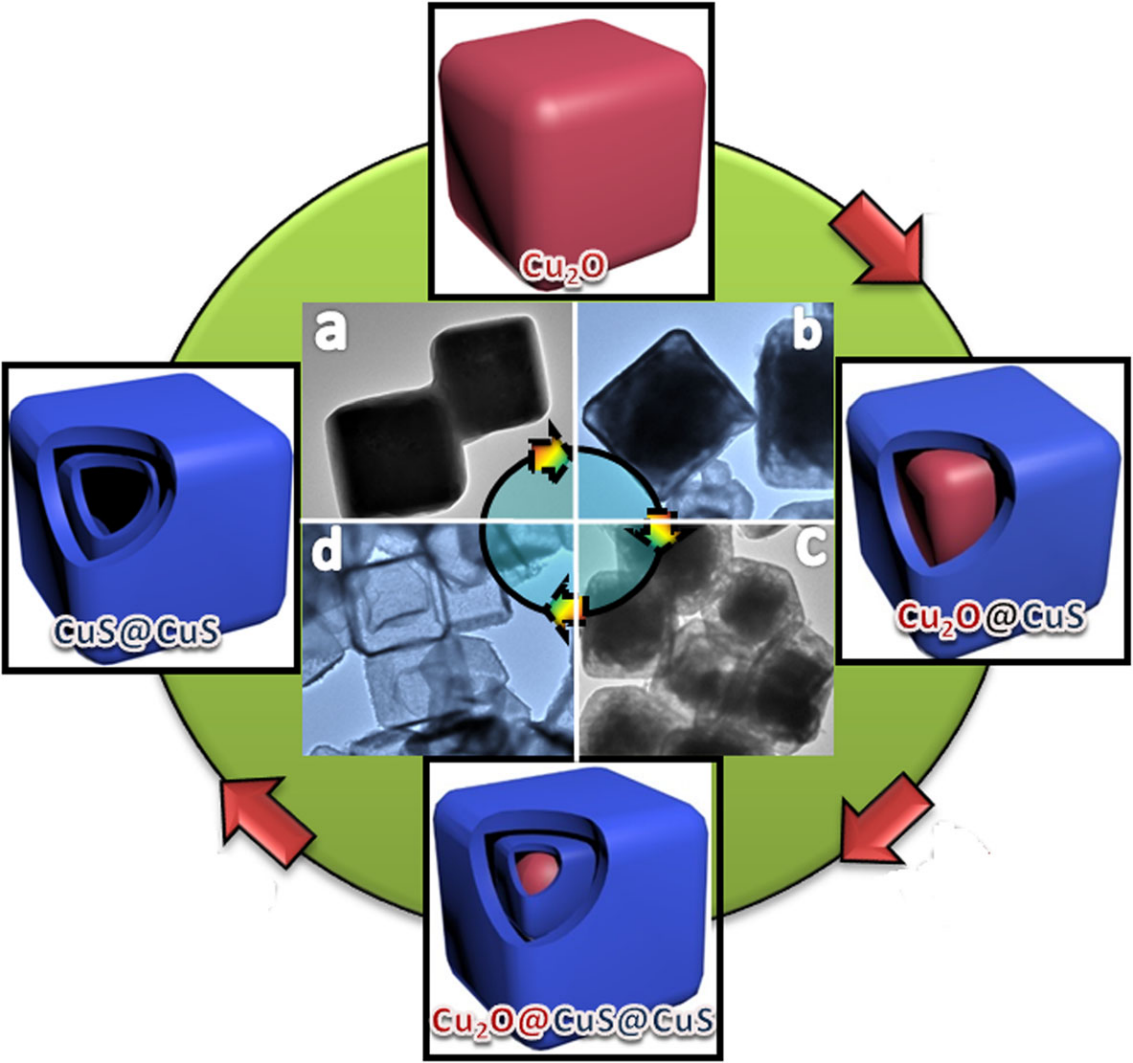
The synthetic process of 2-CuS NCs. The inset represents TEM images matched with **a** Cu_2_O, **b** Cu_2_O@CuS, **c** Cu_2_O@CuS@CuS, and **d** CuS@CuS

Schematic illustration of synthetic process for 2-CuS NCs is depicted in Fig. 1. Firstly, Cu_2_O templates were evenly distributed into the mixed solution of water and alcohol (volume ratio 1:1) under the assistance of ultrasonic. The sulfidation process was driven by S^2−^ ions released from Na_2_S, and a thin layer of CuS was formed around Cu_2_O templates (reaction 1). Then, S_2_O_3_^2−^ ions were introduced and the etching of Cu_2_O occurred (reaction 2) due to soft interaction between Cu^+^ and S_2_O_3_^2−^ [16], resulting in the formation of gap between CuS and Cu_2_O. Afterwards, the above-prepared Cu_2_O@CuS structure was sulfurized for 2 min to generate the inner CuS shell around the residual Cu_2_O templates. Finally, 2-CuS NCs were obtained by complete etching of Cu_2_O templates for 1 h using S_2_O_3_^2−^ ions. The coordinated control of etching rate of Cu_2_O and precipitation of CuS led to the formation of well-defined 2-CuS NCs. TEM images of the products obtained at different stages are also displayed in Fig. 1 (inset a–d). The observed formation process agreed well with the above-deduced mechanism.

1 $$ {\mathrm{Cu}}_2\mathrm{O}+{\mathrm{S}}^{2-}+{\mathrm{O}}_2+4{\mathrm{H}}_2\mathrm{O}\to 4\mathrm{CuS}+8{\mathrm{O}\mathrm{H}}^{-} $$

2 $$ {\mathrm{Cu}}_2\mathrm{O}+{\mathrm{S}}_2{{\mathrm{O}}_3}^{2-}+{\mathrm{H}}_2\mathrm{O}\to {\left[{\mathrm{Cu}}_2\left({\mathrm{S}}_2{{\mathrm{O}}_3}^{2-}\right)x\right]}^{2-2x}+2{\mathrm{O}\mathrm{H}}^{-} $$

**Fig. 2:**
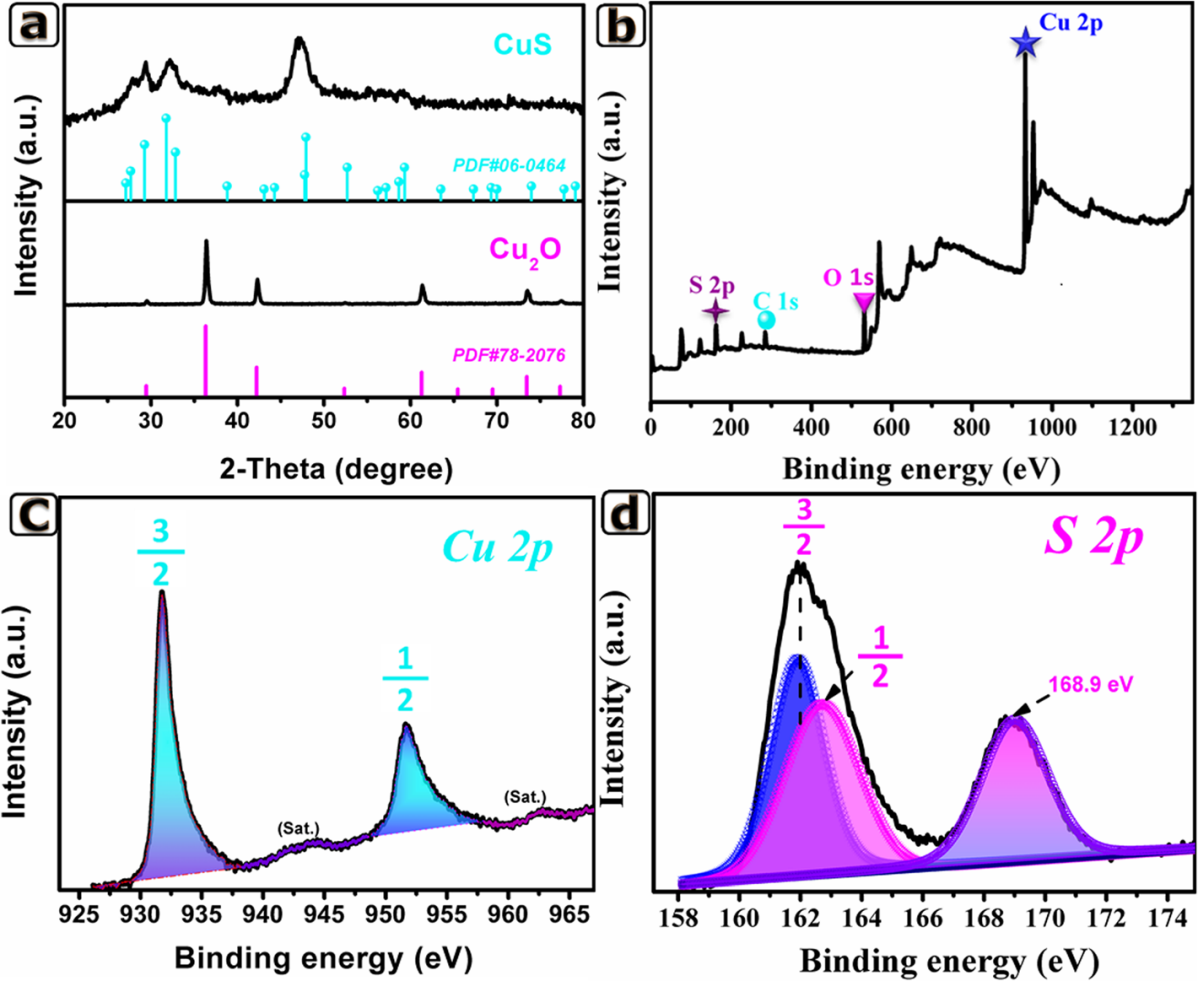
**a** XRD pattern of 2-CuS NCs and Cu_2_O. **b** XPS survey spectrum for the product. **c** Cu 2p. **d** S 2p

As shown in Fig. 2a, all diffraction peaks of final products agreed well with PDF#06-0464, and no diffraction peaks of Cu_2_O was observed, indicating successful preparation of hexagonal CuS. Furthermore, the detailed information on chemical compositions and electronic states of the final products was measured by XPS. The survey spectroscopy demonstrated Cu 2p and S 2p peaks (Fig. 2b), revealing the main composition of the samples. As depicted in Fig. 2c, the two major peaks at 931.8 eV and 951.7 eV were assigned to Cu 2p_3/2_ and Cu 2p_1/2_, respectively. The binding energy separation was about 20 eV, which was the typical characteristic of Cu^2+^ in CuS [8]. Besides, two satellite (Sat.) peaks at 944.1 eV and 962.5 eV were observed in the Cu 2p spectrum, further demonstrating the existence of Cu^2+^ [17]. In the S 2p spectrum (Fig. 2d), the typical peak from 160 to 164 eV was fitted by two peaks located at 161.8 eV and 162.9 eV, which was the feature of S-Cu [8, 18]. A characteristic peak at 168.9 eV also indicated the existence of metal sulfides [19]. The results of XRD and XPS data confirmed the successful preparation of hexagonal CuS.

**Fig. 3:**
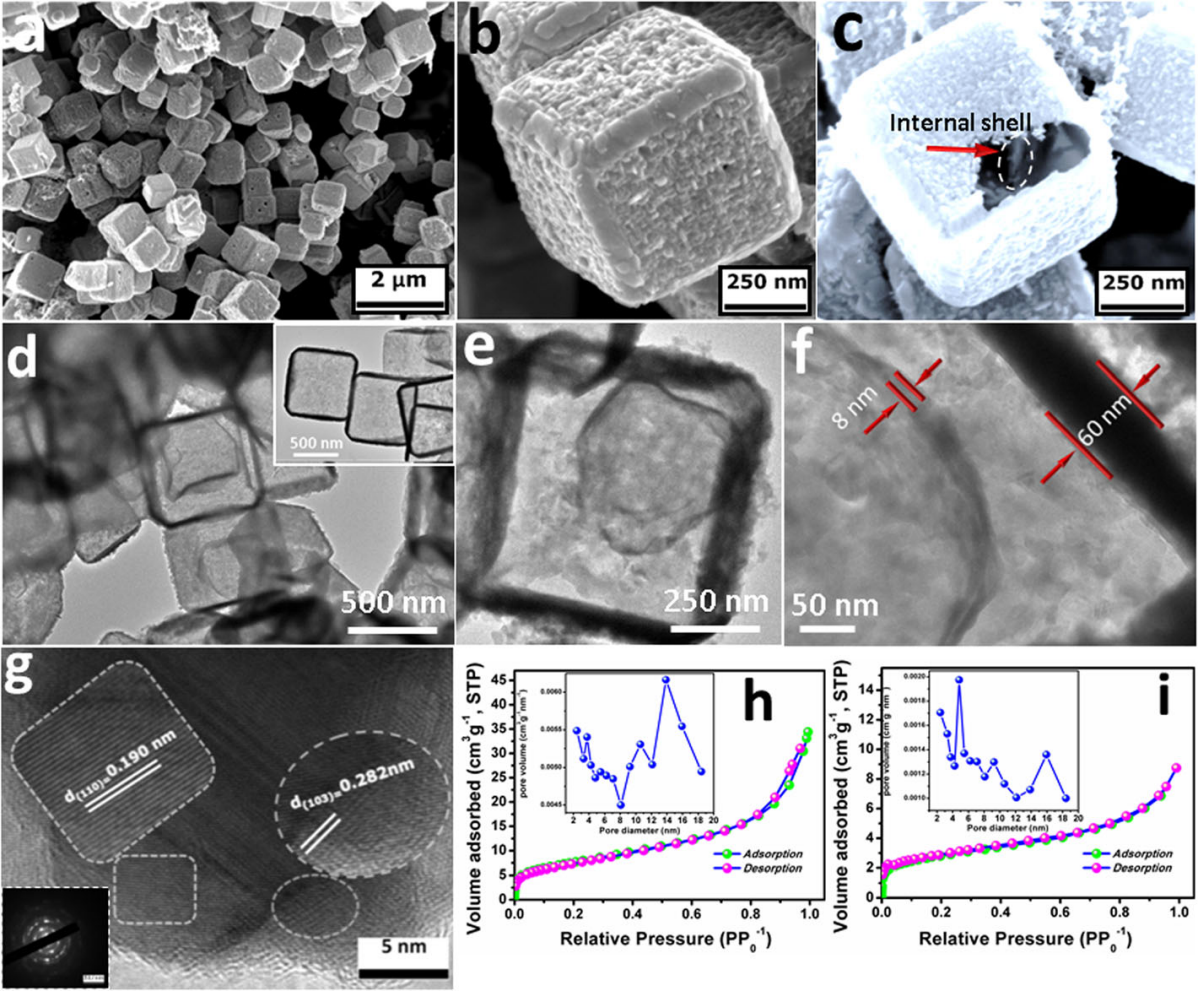
**a**–**c** FESEM images of 2-CuS NCs. TEM images of **d**–**f** 2-CuS NCs and (inset of **d**) 1-CuS NCs. **g** HRTEM image of 2-CuS NCs and insert is the selected area electron diffraction pattern. N_2_ adsorption-desorption isotherm of **h** 2-CuS NCs and **i** 1-CuS NCs. Insets are the corresponding pore size distributions

In Additional file 1: Figure S2, Cu_2_O templates exhibited exquisite cubic morphology with an average edge length about 500 nm. As shown in Fig. 3a, the prepared CuS exactly duplicated structural and morphological features of Cu_2_O templates. The shell of CuS was porous and composed of randomly assembled nanoparticles (Fig. 3b). As shown in Fig. 3c, the broken cube revealed cage-like feature and double-shelled structure of CuS products. The internal CuS NCs further increased the contact area between electrode and electrolyte to provide more electroactive sites, leading to enhanced electrocatalytic activity. The detailed structure of 2-CuS NCs is studied by TEM. As shown in Fig. 3d, the final CuS products presented a typical double-shelled cage-like structure compared with 1-CuS NCs (inset of Fig. 3d). Notably, the inner CuS NCs were not in the central place, and an obvious gap between the two cages was observed (Fig. 3e). As shown in Fig. 2f, the thicknesses of outer and inner shells were about 60 nm and 8 nm, respectively. The decrease of the inside shell thickness can be attributed to the shielding effect of the outside CuS shell. Two distinct lattice fringes of 0.190 nm and 0.282 nm observed in Fig. 3g were consistent with (110) and (103) crystal planes of CuS (PDF#06-0464), respectively. Simultaneously, the selected area electron diffraction pattern in the inset revealed polycrystalline feature of 2-CuS NCs. The results of FESEM and TEM demonstrated the successful preparation of 2-CuS NCs.

To verify the porosity, N_2_ absorption-desorption isothermal and corresponding pore size distributions are recorded in Fig. 3h, i. The curve of 2-CuS NCs was considered as a 4-type isotherm with a H3 hysteresis loop, suggesting the existence of mesopores [20]. The pore size distribution of 2-CuS NCs (inset of Fig. 3h) ranging from 2.4 to 18.5 nm further confirmed the mesoporous feature. Especially, the pore volumes of 2-CuS NCs and 1-CuS NCs were estimated as 0.045 cm^3^ g^−1^ and 0.011 cm^3^ g^−1^, respectively. The mesopores served as suitable channels for ion diffusion and played a key role in facile mass transport during electrocatalytic reactions [21]. Moreover, the surface area of 2-CuS NCs (28.3 m^2^ g^−1^) was much larger than that of 1-CuS NCs (10.03 m^2^ g^−1^). Furthermore, 2-CuS NCs also had larger surface area compared with the previously reported CuS materials, including nanosheets [22], nanoplates [23], nanoflowers [24], and nanospheres [25]. Generally, high porous volume and large surface areas benefited the accessibility of reactant molecules to the inner shells of 2-CuS NCs, leading to enhanced electrocatalytic activity.

### Electrochemical Performance of 2-CuS NCs/GCE

**Fig. 4:**
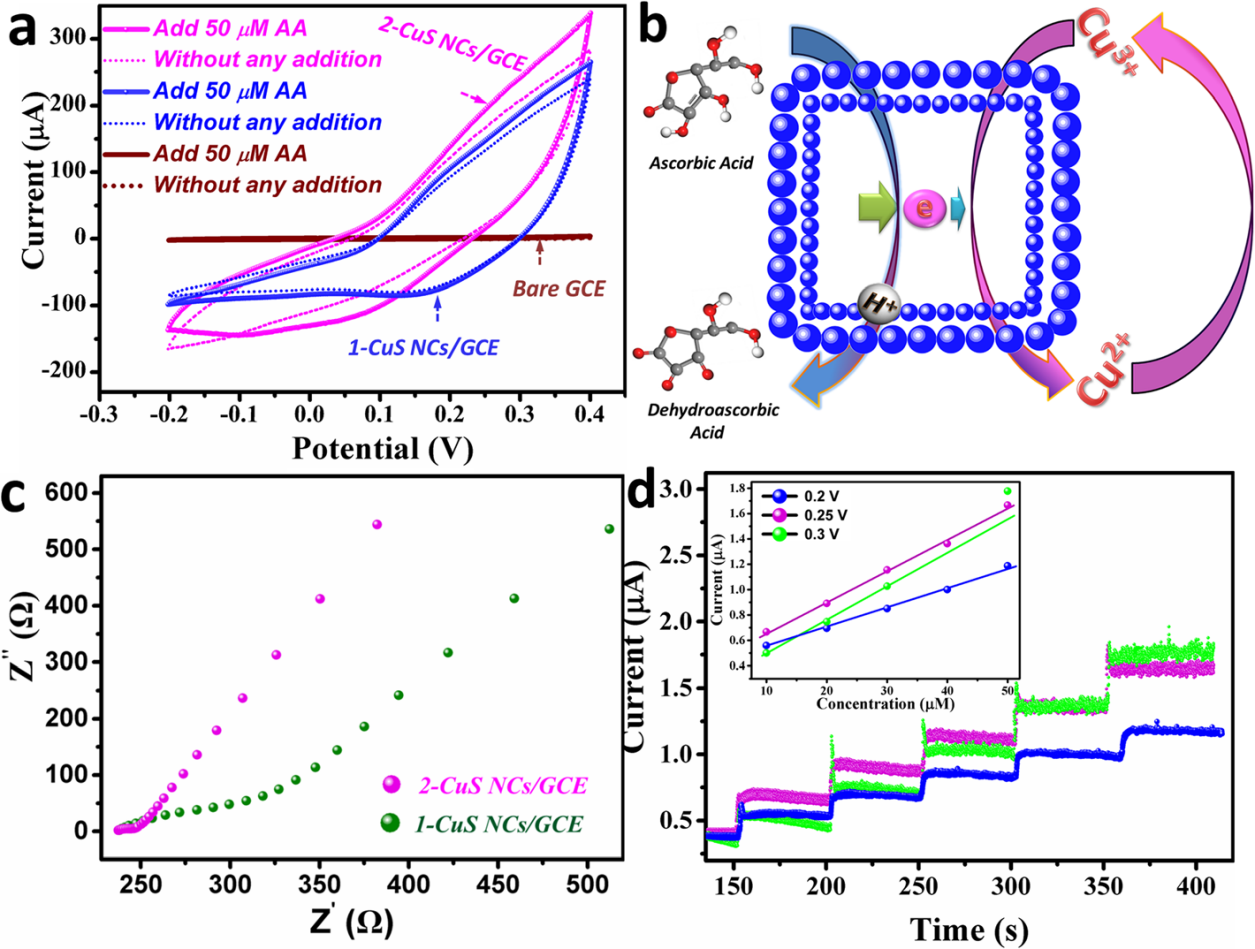
**a** CVs of 2-CuS NCs/GCE, 1-CuS NCs, and bare GCE at 50 mV s^−1^. **b** The catalytic mechanism of AA oxidation on 2-CuS NCs/GCE. **c** Nyquist plot of 2-CuS NCs/GCE and 1-CuS NCs/GCE. **d** *i*-*t* response towards 25-μM AA

Cyclic voltammetry (CV) was performed to study the electrocatalytic activity of 2-CuS NCs/GCE towards AA. Figure 4a displays the CVs of bare GCE, 1-CuS NCs/GCE, and 2-CuS NCs/GCE in the absence and presence of 50 μM AA. Obviously, bare GCE had a small background current, whereas the modified GCE had much better conductivity compared with bare GCE. After the addition of 50 μM AA, extremely weak current response was investigated on bare GCE (Additional file 1: Figure S3). However, current responses were clearly observed for another two electrodes. Remarkably, 2-CuS NCs/GCE exhibited higher current response than that of 1-CuS NCs/GCE, revealing higher electrocatalytic activity. The active redox couple of Cu^2+^/Cu^3+^ plays a crucial role in AA oxidation [14], and the catalytic mechanism on 2-CuS NCs/GCE is discussed in Fig. 4b. Firstly, Cu got a high oxidizing state due to the initial conversion of Cu^2+^ to Cu^3+^. Then, AA molecules enriched on the surface of 2-CuS NCs/GCE were oxidized to dehydroascorbic acids by Cu^3+^, while Cu^3+^ obtained electrons from AA and reduced to low valence state of Cu^2+^.

In order to study the kinetics advantages, electrochemical impedance spectroscopy (EIS) was recorded. As shown in Fig. 4c, the Nyquist plots were consisted of a semicircle portion in high frequency and a linear portion in low frequency. The semicircle corresponded to the electron transfer resistance, and the linear portion was related to the ion diffusion resistance. Obviously, 2-CuS NCs/GCE showed a smaller semicircle than that of 1-CuS NCs/GCE, revealing lower electron transfer resistance. The lower electron transfer resistance can be ascribed to the high electron collection efficiency and elevated electron transfer rate provided by the double-shelled structure. Notably, the slope in the low frequency region along the imaginary axis for 2-CuS NCs/GCE was subvertical, demonstrating a low ion diffusion resistance issued from enhanced porosity of the shells and interior cavities [18, 26].

In Additional file 1: Figure S4, the effect of scan rates on CVs of 2-CuS NCs/GCE was recorded. The redox peak current linearly changed with square root of scan rates (inset), indicating a diffusion-controlled process on the surface of 2-CuS NCs/GCE [27]. Additional file 1: Figure S5a and Figure S5b displayed the chronoamperometry (CA) response for 1-CuS NCs/GCE and 2-CuS NCs/GCE in 0 mM and 0.5 mM of AA at 0.25 V. In a static AA solution, a large diffusion current was produced once the potential was applied in the CA because of the high concentration gradient. Then, the diffusion current gradually decreased with the decrease of concentration gradient. Finally, a stable diffusion current was maintained due to the stable diffusion of AA from solution to electrode. The diffusion coefficient (*D*) of AA can be calculated according to Cottrell’s equation [28]:

3 $$ I\mathrm{cat}=\mathrm{nF}A{D}^{1/2}C0{\pi}^{-1/2}{t}^{-1/2} $$

where *I*_cat_ is the current of the electrode in 0.5 mM AA, *n* represents the number of electrons transferred, *F* is the Faraday constant, *A* is the area of the electrode, *C*_*0*_ is the substrate concentration, *D* is the diffusion coefficient, and *t* expresses the elapsed time. Additional file 1: Figure S5c showed the plots of *I*_cat_ vs *t*^−1/2^ according to the CA curves. Thus, the value of *D* for 2-CuS NCs/GCE could be calculated to be 2.77 × 10^−5^ cm^2^ s^−1^, which was larger than 1-CuS NCs/GCE (4.16 × 10^−7^ cm^2^ s^−1^). The catalytic rate constant (*K*_cat_) of AA oxidation can be calculated according to the following equation:

4 $$ {I}_{\mathrm{cat}}/{I}_L={\left(\pi {k}_{\mathrm{cat}}{C}_{0t}\right)}^{1/2} $$

where *I*_cat_ and *I*_L_ are the diffusion currents of the electrode in 0.5 mM and 0 mM AA, respectively. *C*_*0*_ is the substrate concentration, and *t* is the elapsed time. According to Additional file 1: Figure S5d, the value of *K*_cat_ was estimated to be 0.08 × 10^3^ M^−1^ s^−1^, which was larger than that of 1-CuS NCs/GCE (0.02 × 10^3^ M^−1^ s^−1^). Generally, the elevated values of *D* and *K*_cat_ would result in higher electrocatalytic activities.

### Detection of AA

**Fig. 5:**
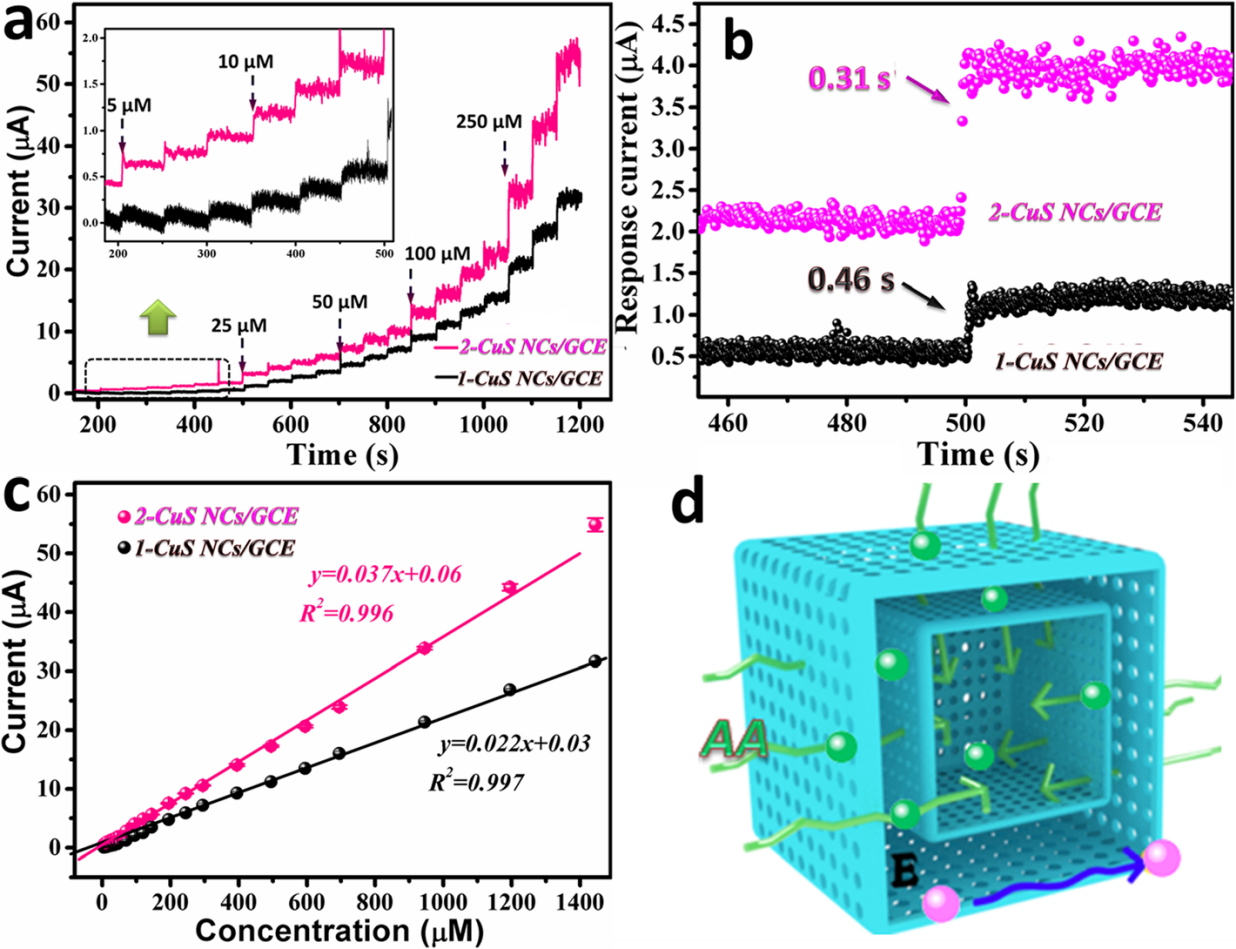
**a** *i*-*t* response at different working potentials. **b** *i*-*t* response of 2-CuS NCs/GCE and 1-CuS NCs/GCE at 0.25 V. **c** The corresponding calibration plot of **b**. **d** The illustration of dynamic advantages for 2-CuS NCs

To obtain the optimal working potential, *i*-*t* curves at different potentials are gathered in Fig. 4d. Obviously, the current response at 0.25 V was higher than that at 0.2 V, and the relationship between concentration and response current at 0.25 V showed a better linearity than 0.3 V (inset of Fig. 4d). Moreover, severe interference to the oxidation of AA easily emerged at more positive potential, so 0.25 V was selected as the optimal working potential. As demonstrated in Fig. 5a, 2-CuS NCs/GCE exhibited superior amperometric response to 1-CuS/GCE. Once AA was added to the electrolyte, the response current immediately reached 95% of the steady-state current within 0.31 s for 2-CuS NCs/GCE and 0.46 s for 1-CuS NCs/GCE (Fig. 5b), suggesting that 2-CuS NCs/GCE had faster response towards AA. As shown in Fig. 5c, the response current linearly increased with AA concentrations between 5 and 1200 μM, and the regression equation was expressed as *I* (μA) = 0.037C (μM) + 0.06 (*R*^2^ = 0.996). The sensitivity was calculated as 523.7 μA mM^−1^ cm^−2^, which was higher than that of 1-CuS/GCE (324.4 μA mM^−1^ cm^−2^). Furthermore, the 2-CuS NCs/GCE presented a LOD as low as 0.15 μM at signal-to-noisy ratio of 3. The enhanced electrocatalytic performance of 2-CuS NCs can be attributed to the coupling of two hollow structures (Fig. 5d). (1) Larger surface areas and more active sites were acquired to improve redox reactions. This point was proved by BET analysis; (2) larger volume occupying rate and ample mesopores effectively promoted the utilization of the double-shelled cage-like structure; (3) the two thin shells of 2-CuS NCs accelerated the transfer rate of catalytic electron, which was confirmed by above EIS analysis. Compared with the previously reported literatures, 2-CuS NCs/GCE exhibited higher electrochemical performance in terms of high sensitivity and low LOD as shown in Table 1 [29–35], demonstrating that 2-CuS NCs was ideal for analytical sensing of AA.

**Table 1:** Comparison of 2-CuS NCs/GCE with previously reported nonenzymatic AA sensors

Electrodes	Linear range (mM)	Sensitivity (μA mM^−1^ cm^2^)	LOD (μM)	Reference
EMGON 5-1/CPE	0–1	78.63	1.54	[29]
Au-PANI/GCE	0.01–12	25.69	8.2	[30]
CF/ZnO/GCE	0.6–1.8	–	156.7	[31]
Ag HN/GCE	0.00017–1.8	35.512	0.06	[32]
GNP-Ag/GCE	0.001–0.2	–	0.88	[33]
RO/Ni	0.57–5.7	296	–	[34]
Bi_2_S_3_/TNF/GCE	1–10	38	–	[35]
2-CuS NCs/GCE	0.005–1.2	523.7	0.15	This work

*EMGON* electroactive mesoporous materials organosilica nanocomposites, *CPE* carbon paste electrode, *Au-PANI* polyaniline, *CF* carbon fiber, *HN* hierarchical nanostructures, *GNP* graphene nanoplatelets, *RO* ruthenium oxide, *TNF* titanate nanofibers

### Selectivity, Reproducibility, and Stability of the 2-CuS NCs/GCE

**Fig. 6:**
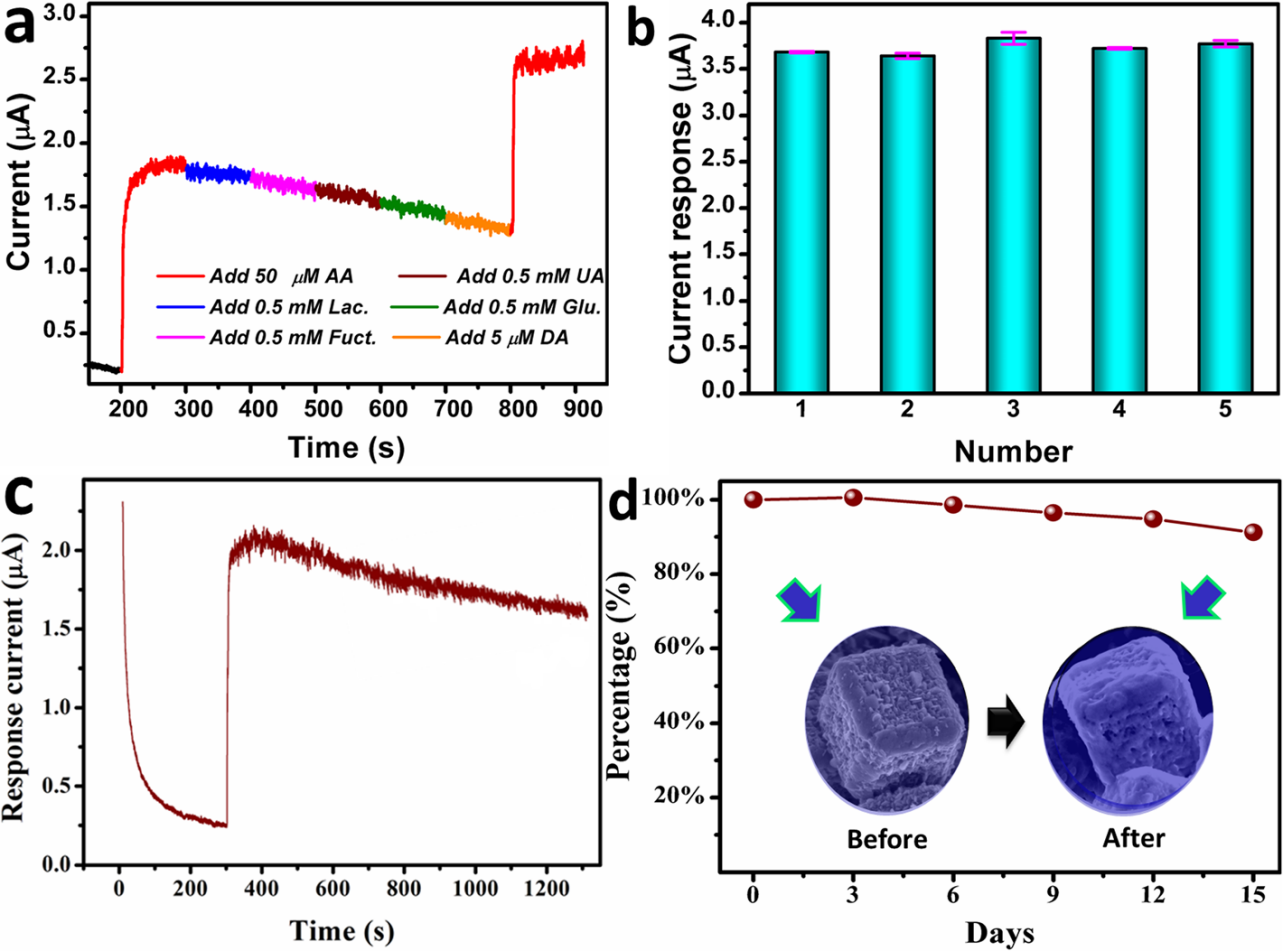
**a** CA of 2-CuS NCs/GCE with successive addition of different species. **b** Current responses of five 2-CuS NCs electrodes towards 100-μM AA. **c** The stability of 2-CuS NCs/GCE with running time. **d** Long-term stability of 2-CuS NCs/GCE. Inset is the FESEM images of 2-CuS NCs/GCE before and after electrochemical detection

The selectivity, reproducibility, and stability were also of great importance in electrochemical sensing of AA. The common interfering species were injected during the *i*-*t* measurement to evaluate the selectivity. As shown in Fig. 6a, no significant interference currents were observed, indicating ultrahigh selectivity. Moreover, the response current for the second addition of AA still retained 91% of its first injection. The attenuation in response current would be ascribed to the adsorption of trace interfering species or intermediate products on the electrode. As depicted in Fig. 6b, the response current of five different electrodes towards 100 μM AA was recorded, and the relative standard deviation (RSD) was 3.6%, suggesting good reproducibility. In terms of long-term stability, only 15% of the current response was lost over a long period of 1000 s (Fig. 6c). As shown in Fig. 6d, the response current of 2-CuS NCs/GCE still retained 91.2% of the initial value after 15 days. Moreover, 2-CuS NCs still maintained cubic structure after testing (inset), demonstrating remarkable stability. The excellent stability could be ascribed to the double-shelled highly porous feature, which alleviated the structure strain associated with the volume expansion during electrochemical testing.

## Conclusions

In brief, we have succeeded in preparation and application of 2-CuS NCs in enzyme-free AA electrochemical sensor. Optimized double-shelled cage-like structure for CuS NCs afforded large specific surface area, increased volume occupying rate, enough diffusion channels, and confined electron transfer routes, leading to prominent electrocatalytic activity. The unique structure yielded 2-CuS NCs/GCE with short response time (0.31 s), high sensitivity (523.7 μA mM^−1^ cm^−2^), low LOD (0.15 μM), reasonable selectivity, and acceptable reproducibility towards AA. Overall, 2-CuS NCs look promising as effective electrocatalysts for electrochemical sensing of AA.

## Supplementary information

**Additional file 1:** **Figure S1.** (a) FESEM image and (b) TEM image of 1-CuS NCs. **Figure S2.** (a) FESEM image of Cu_2_O. **Figure S3.** CVs of bare GCE at 50 mV s^-1^. **Figure S4.** CVs of 50 μM AA on 2-CuS NCs/GCE at different scan rates (16, 25, 36, 49, 64, 81 and 100 mV s^-1^). **Figure S5.** Chronoamperograms of (a) 2-CuS NCs/GCE and (b) 1-CuS NCs/GCE in the absence and presence of 0.5 mM AA; (c) Calibration curve of *I*_cat_ versus *t*^*-1/2*^; (d) Calibration curve of *I*_cat_*/I*_*L*_ versus *t*^*1/2*^.

## Data Availability

The datasets are available without restriction.

## Abbreviations

1-CuS NCs: Single-shelled CuS nanocages
2-CuS NCs: Double-shelled CuS nanocages
AA: Ascorbic
AA: l-ascorbic acid
BET: Brunauer-Emmett-Teller
CA: Chronoamperometry
CV: Cyclic voltammetry
DA: Dopamine
EIS: Electrochemical impedance spectroscopy
FESEM: Field emission scanning electron microscope
Fruc: Fructose
GCE: Glassy carbon electrode
Glu: Glucose
HRTEM: High-resolution transmission electron microscope
Lac: Lactose
LOD: Limit of detection
PBS: Phosphate solution
Sat.: Satellite
UA: Uric acid
XPS: X-ray photoelectron spectroscopy
XRD: X-ray diffraction
