# Supplementary material for: Design of Double-Shelled CuS Nanocages to Optimize Electrocatalytic Dynamic for Sensitive Detection of Ascorbic Acid
Source: Nanoscale Res Lett. 2020 Feb 18;15:44. doi: 10.1186/s11671-020-3278-2 (PMC7028898; doi:10.1186/s11671-020-3278-2)
Supplement: Supplementary file 1 — Additional file 1: Figure S1. (a) FESEM image and (b) TEM image of 1-CuS NCs. Figure S2. (a) FESEM image of Cu2O. Figure S3. CVs of bare GCE at 50 mV s-1. Figure S4. CVs of 50 μM AA on 2-CuS NCs/GCE at different scan rates (16, 25, 36, 49, 64, 81 and 100 mV s-1). Figure S5. Chronoamperograms of (a) 2-CuS NCs/GCE and (b) 1-CuS NCs/GCE in the absence and presence of 0.5 mM AA; (c) Calibration curve of Icat versus t-1/2; (d) Calibration curve of Icat/IL versus t1/2. [file 11671_2020_3278_MOESM1_ESM.docx]

**Design of double-shelled CuS Nanocages to Optimize Electrocatalytic Dynamic for Sensitive Detection of Ascorbic Acid**

Tong Yang^1,3,4^, Liangliang Tian^2,3,4*^, Enmin Zhou^5^, Daidong Chen^1,3,4^ and Yu Lei ^2,3,4*^

^1^Faculty of Materials and Energy, Southwest University, Chongqing, PR China

^2^Research Institute for New Materials Technology, Chongqing University of Arts and Sciences, Chongqing, PR China

^3^Chongqing Key Laboratory of Materials Surface and Interface Science, Chongqing, PR China

^4^Chongqing Municipal Key Laboratory of Micro/Nano Materials Engineering and Technology, Chongqing, PR China

^5^School of Science, Chongqing University of Posts and Telecommunication, Chongqing, PR China

^*^Corresponding author.

E-mail addresses: tianll07@163.com (LL Tian)


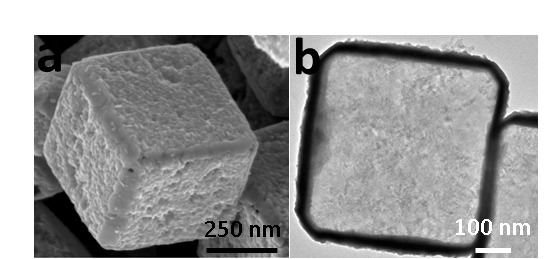


Fig. S1. (a) FESEM image and (b) TEM image of 1-CuS NCs.


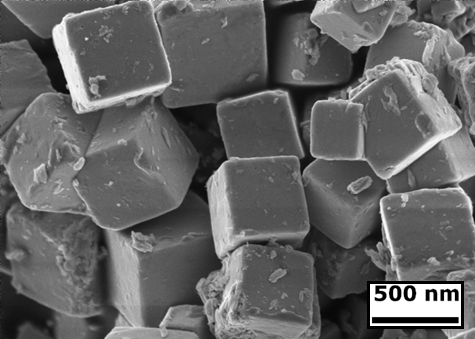


Fig. S2. (a) FESEM image of Cu_2_O


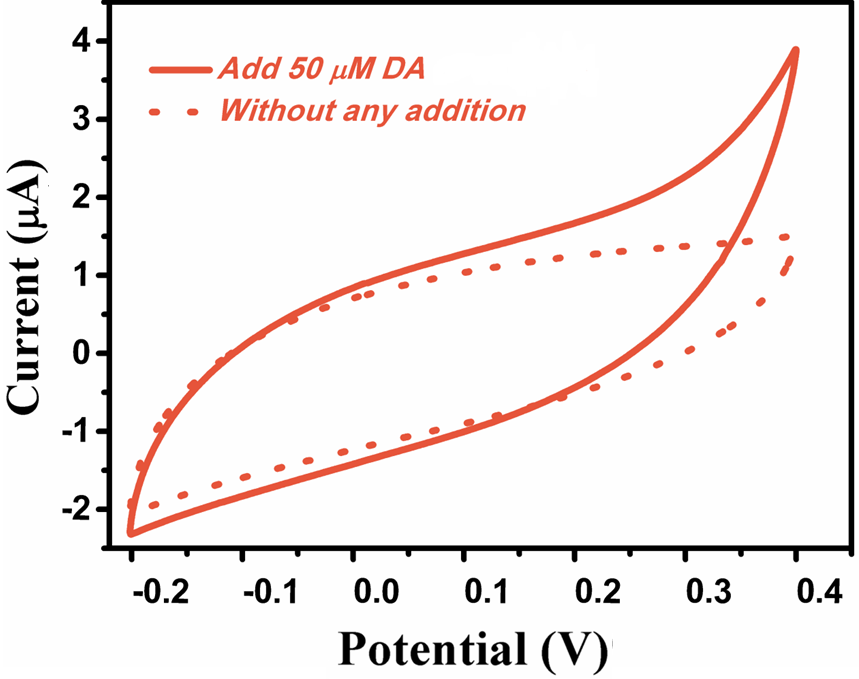


Fig. S3. CVs of bare GCE at 50 mV s^-1^.


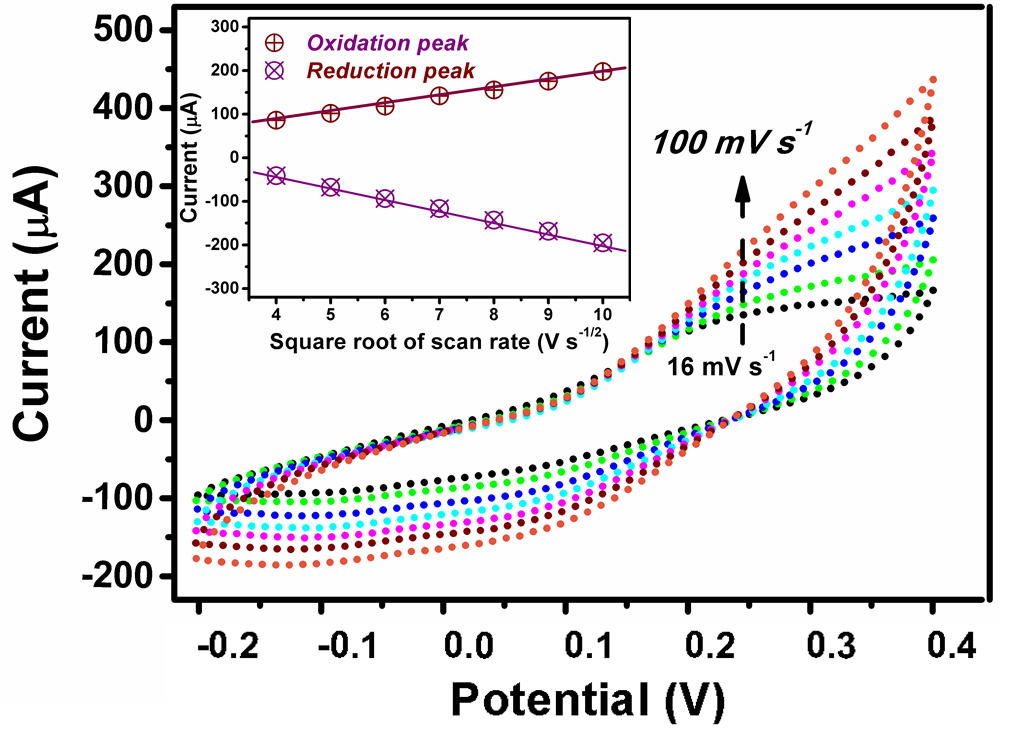


Fig. S4. CVs of 50 μM AA on 2-CuS NCs/GCE at different scan rates (16, 25, 36, 49, 64, 81 and 100 mV s^-1^)


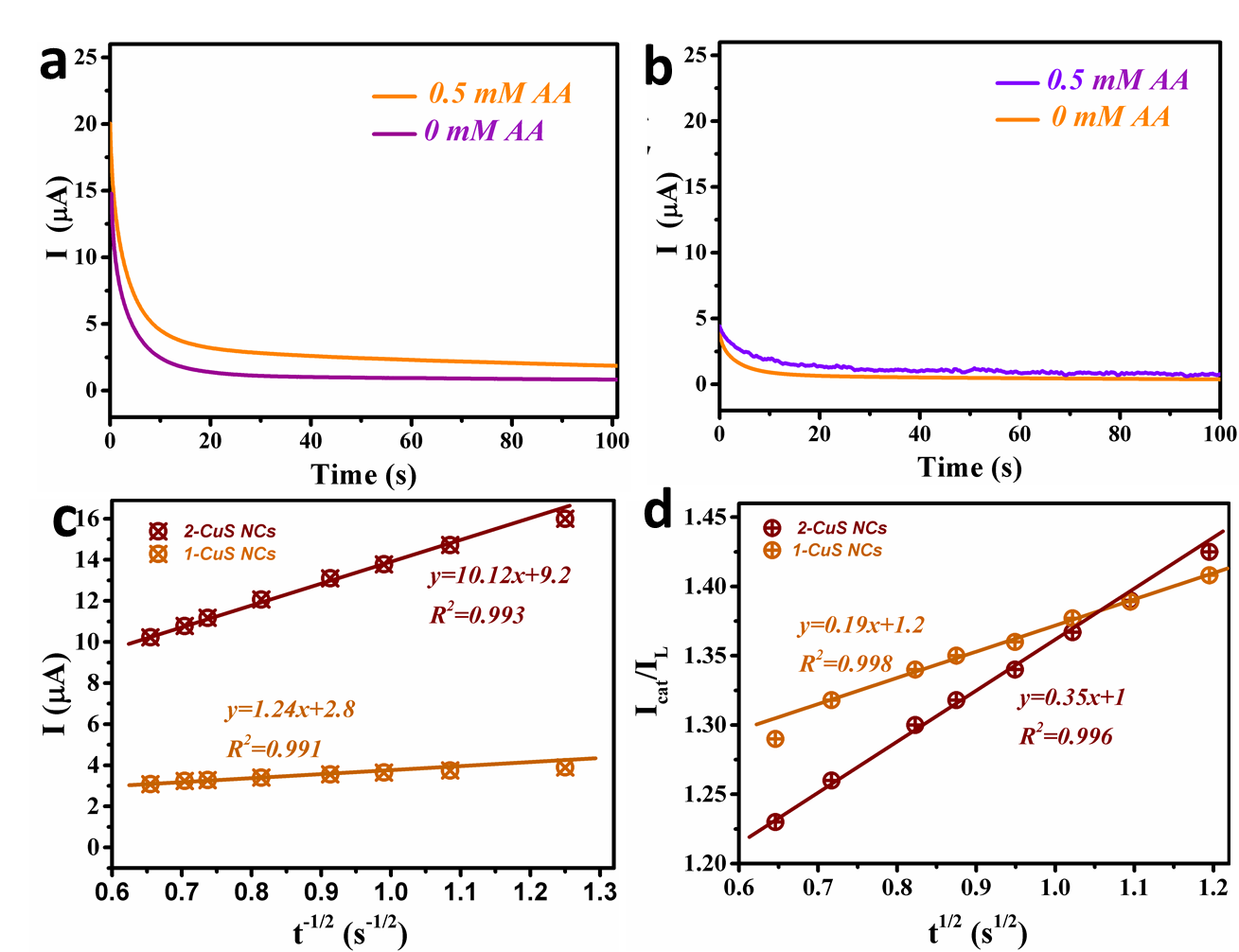


Fig. S5. Chronoamperograms of (a) 2-CuS NCs/GCE and (b) 1-CuS NCs/GCE in the absence and presence of 0.5 mM AA; (c) Calibration curve of *I_ca_*_t_ versus *t^-1/2^*; (d) Calibration curve of *I_cat_/I_L_* versus *t^1/2^*.
